# Stem cell treatment for type 1 diabetes

**DOI:** 10.3389/fcell.2014.00009

**Published:** 2014-03-20

**Authors:** Ming Li, Susumu Ikehara

**Affiliations:** Department of Stem Cell Disorders, Kansai Medical UniversityHirakata City, Osaka, Japan

**Keywords:** type 1 diabetes, tissue-derived-stem cell, autoimmune disease, bone marrow transplantation, autoantibodies

## Abstract

Type 1 diabetes mellitus (T1DM) is a common chronic disease in children, characterized by a loss of β cells, which results in defects in insulin secretion and hyperglycemia. Chronic hyperglycemia causes diabetic complications, including diabetic nephropathy, neuropathy, and retinopathy. Curative therapies mainly include diet and insulin administration. Although hyperglycemia can be improved by insulin administration, exogenous insulin injection cannot successfully mimic the insulin secretion from normal β cells, which keeps blood glucose levels within the normal range all the time. Islet and pancreas transplantation achieves better glucose control, but there is a lack of organ donors. Cell based therapies have also been attempted to treat T1DM. Stem cells such as embryonic stem cells, induced pluripotent stem cells and tissue stem cells (TSCs) such as bone marrow-, adipose tissue-, and cord blood-derived stem cells, have been shown to generate insulin-producing cells. In this review, we summarize the most-recently available information about T1DM and the use of TSCs to treat T1DM.

## Introduction

Type 1 diabetes mellitus (T1DM) is a T cell-mediated autoimmune disease, induced by permanent destruction of β cells. Hyperglycemia causes complications that include diabetic nephropathy, neuropathy, and retinopathy in T1DM patients. Insulin therapy is effective because insulin is deficient in T1DM patients. Available insulin delivery systems include syringes, pumps, jet injectors, and pens. Insulin therapy helps decrease blood glucose levels, but does not maintain the levels in the normal range over extended periods. β cell replacement therapies, including islet and pancreas transplantation, have been shown to be a useful approach to T1DM, but this approach suffers from a lack of donors. Thus, stem cells therapies have recently been in the spotlight as a means of controlling T1DM. Stem cells include induced pluripotent stem cells (iPS), embryonic stem cells (ESCs), and tissue-derived-stem cells, such as bone marrow-, adipose-, and cord blood-derived-stem cells (Hussain and Theise, 2004). This review looks at the use of tissue-derived-stem cells for the treatment of T1DM.

## Pathophysiology of T1DM

T1DM is an autoimmune disorder in which β cells are destroyed by immunoresponse. A T1DM animal model, the non-obese diabetic (NOD) mouse, has many of the same autoantigens targeted by human T cells (Delovitch and Singh, 1997; Atkinson and Leiter, 1999). NOD mice, which are genetically deficient in B lymphocytes, develop a very low incidence of diabetes, one report suggesting that depleting B lymphocytes delays and/or reduces the onset of diabetes (Hu et al., 2007).

Major histocompatibility complex (MHC) class I and II-restricted islet-antigen-reactive T cells have been detected in the NOD mouse, the NOD MHC I-A^g7^ allele being essential for disease development (Bluestone et al., 2010). More than 20 potential *ldd* loci were found to affect the development of T1DM, including T lymphocyte antigen 4, and IL-2. Female NOD mice show a higher incidence of diabetes than male mice, but another T1DM animal model, the inbred BioBreeding (BB) rat, shows no difference between the sexes in the incidence of T1DM, its MHC gene product being RT1^u/u^. Further, more than 12 loci related to the development of diabetes have been detected. Some autoantigens, including insulin, glutamic acid decarboxylase (GAD) 65, IGRP, IA-2 and IA-β (phogrin) have been detected in T1DM (Lieberman and DiLorenzo, 2003). CD4^+^, CD8^+^ T cells, and macrophages have a role in the death of β cells. Dendritic cells (DCs), natural-killer (NK) cells and NKT cells have been shown to contribute to β cell death (Lehuen et al., 2010). CD4^+^T cells play an important role in both the early and late stages of T1DM. CD8^+^ T cells, which infiltrate the islets of NOD mice, recognized the islet-specific glucose-6-phosphatase catalytic subunit-related protein (IGRP); when IGRP autoimmunity was prevented, so was the development of diabetes (Han et al., 2005a,b). Regulatory T cells (T reg) play an important role in autoimmune diabetes, their number, and function changing in the pancreas of autoimmune mice. The number of IFNγ-producing T reg cells is significantly lower in the peripheral blood of T1DM patients (D'Alise et al., 2008; Tang et al., 2008). Macrophages produce IL-12 to promote CD8+ differentiation, and produce IL-1β, TNF, and ROS to cause β cell death. NK cells were found to infiltrate the pancreas and directly or indirectly destroy β cells (Feuerer et al., 2009). Macrophages, DCs, and NK cells produce inflammation cytokines such as IFN-α and IFN-γ, which damage β cells in the pancreata, and the NK cells also destroy the β cells when there is a viral infection (Fairweather and Rose, 2002). Environmental factors also strongly affect the progression of T1DM. For example, the incidence of diabetes decreased when mice were exposed to microbial stimuli (Wen et al., 2008).

Abnormal T cells infiltrate the islets and destroy the β cells because they do not recognize β cell antigens as self antigens. T cell precursors in the bone marrow (BM) develop into mature T cells by positive and negative selection in the thymus and then migrate to the peripheral tissue (Heinzel et al., 2007). Thymocytes expressing low-affinity TCRs (T-cell receptors) populate the peripheral lymphoid organs, where they can recognize foreign antigens. Autoreactive T cells can escape thymocyte negative selection and elicit autoimmunity in the absence of adequate peripheral regulation (Marrack and Parker, 1994; Han et al., 2005a,b). Approximately 20% of individuals with spontaneous mutation of autoimmune gene Aire develop T1DM with other autoimmune diseases, which reflects their inability to select against islet antigen reactivity (Gardner et al., 2009).

T1DM is a highly multigenic autoimmune disease in humans, and some autoantibodies have been detected in the peripheral blood after the onset of diabetes. Autoantigens such as insulin, Glutamate decarboxylase (GAD) 65, islet antigen (IA)-2 and IGRP were defined as recognized by T cells in T1DM patients (Yamamoto et al., 2004). The increased proliferation of CD4+ T cells has been reported in the presence of GAD extracted from human brain and islets (Harrison et al., 1993). Autoantigen-specific CD4+T cells have been studied in very different clinical settings, including T1DM patients undergoing pancreas/kidney transplantation. Autoantibodies were detected pre-transplant or reappeared post-transplant in normoglycemic patients (Vendrame et al., 2010). And a strong inverse correlation has been found to exist between the binding affinity of β cell peptides to HLA-A and CTL responses against those peptides in recently-diagnosed T1DM patients. These data confirmed that many β cell epitopes are recognized by CTLs. Moreover, pathogenic CD8+T cells target HLA-A*0201 in transgenic NOD mice (Takaki et al., 2006).

## Therapies for T1DM

Insulin plays a key role in controlling hyperglycemia in T1DM patients, and the available methods of delivery include syringes (Keith et al., 2004), pumps and jet injectors (Keith et al., 2004) and pens (Wong et al., 2013). Insulin therapy reduces microvascular risk in T1DM patients (Writing Team for the Diabetes Control and Complications Trial/Epidemiology of Diabetes Interventions and Complications Research Group, 2002). But although hyperglycemia can be improved by insulin administration, exogenous insulin injection cannot exactly replicate the insulin secretion from normal β cells when the blood glucose level constantly changes. Islet and pancreas transplantation are more effective treatments, but there is a lack of donors (Weir et al., 2011). Recently, researchers have focused on the generation of new β cells from stem cells for the treatment of T1DM, and this may be one of the most significant advances in the treatment of this disease (Weir et al., 2011). T1DM patients tend to have decreased numbers of endothelial progenitor cells with reduced repair potential, and these fail to differentiate into functional vasculatures (Caballero et al., 2007). However, one report has indicated that diabetes was improved after purified BM endothelial progenitors were transplanted into diabetic mice, suggesting that repairing islet vascularity helped preserve the newly-formed β cells (Wan et al., 2013).

In addition to insulin therapy, a number of approaches, such as the use of drugs and anti-cytokines, have been tested for treating T1DM, and some have been or are in clinical trials. One trial assessed the effects of mucosal insulin therapy for primary immunoprevention (Bonifacio et al., 2008). Another found that the combination of the immunosuppressant drugs mycophenolate mofetil and daclizumab did not preserve β cell function or decrease insulin requirements in T1DM patients (Gottlieb et al., 2010). In the case of Rituximab, another drug that targets the CD20 transmembrane receptor expressing on B lymphocytes, there was no significant difference between patients treated with this drug and placebo-treated groups (Pescovitz et al., 2009). Also, anti-TNF-α therapy failed to prevent the development of T1DM (Koulmanda et al., 2012), but the inhibition of IL-1 action does have clinical efficacy in many inflammatory diseases. The blockade of IL-1 action reduced the incidence of T1DM in animals, and clinical trials have been started to study the feasibility, safety and efficacy of IL-1 therapy in T1DM patients (Tack et al., 2009; Mandrup-Poulsen et al., 2010). The blockade of IL-1β also modulated the effects of anti-CD3 antibody, and the combination of anti-CD3 antibody with IL-1 receptor antagonist thus improved islet inflammation and reversed diabetes in NOD mice (Ablamunits et al., 2012). An antigen-based therapy, alum-conjugated glutamic acid decarboxylase immunization (GAD-Alum), has been reported to successfully treat T1DM in a pilot study, but failed to alter the course of loss of insulin secretion during a 1 year study of patients with recently diagnosed T1DM (Ludvigsson et al., 2008; Wherrett et al., 2011).

## Stem cell treatment for diabetic animals

ESCs, iPS cells and BM-, liver- and pancreas-derived stem cells can differentiate into β cells. Hepatic stem cells expressing duodenum homeobox protein-1 differentiate into β cells, and improved hyperglycemia in diabetic mice (Yang, 2006). ESCs are isolated from blastocysts, and can differentiate into endoderm, mesoderm, and ectoderm cells. They can also differentiate into insulin-producing cells (Soria et al., 2000), and these were able to release insulin in response to glucose stimuli and to normalize the blood glucose levels in diabetic mice when transplanted into those mice (Naujok et al., 2008). iPS was induced from mouse embryonic and adult fibroblast cultures by introducing four factors (Oct3/4, Sox2, c-Myc, and Klf4), and the transplantation of iPS cells corrected hyperglycemia in a T1DM mouse model (Takahashi and Yamanaka, 2006; Alipio et al., 2010). Although ESCs are pluripotent stem cells and can generate insulin-positive cells *in vitro*, *in vitro* differentiation cannot be controlled (Segev et al., 2004; Brolen et al., 2005).

BM mainly includes hematopoietic stem cells (HSCs), which differentiate into myeloid and lymphoid lineages, and mesenchymal stem cells (MSCs), which can differentiate into myogenic, osteogenic, chondrogenic, and adipogenic lineages (Pittenger et al., 1999; Colter et al., 2000). BM cells have the ability to differentiate *in vivo* into functionally competent β cells (Ianus et al., 2003), and NOD mice allotransplantated with BALB/c nu/nu BM cells displayed normal T- and B-cell functions, and newly developed T cells were tolerant to both donor and host. These results suggest that allogeneic bone marrow transplantation (ABMT) might prevent islet destruction, and help to restore self-tolerance (Ikehara et al., 1985). One report has indicated that BMT promotes β cell regeneration after acute injury through BM mobilization (Hasegawa et al., 2007). MSCs are also multipotent cells that can be isolated from not only BM but also adipose tissue and cord blood. MSCs have significantly induced T-reg cells, suppressed β cell-specific T cell proliferation in the pancreas, and overcome the inherent autoimmune pathology associated with T1DM (Urban et al., 2008; Madec et al., 2009). More recently, one report has shown that mouse MSCs can differentiate into insulin-producing cells through recombinant lentiviral transduction of the pdx-1 gene *in vitro* (Rahmati et al., 2013).

## Clinical application for T1DM

A case report on the transplantation of allogeneic amniotic stem cells (high percentage of CD34+ cells) in a young T1DM patient, suggests that hyperglycemia had been improved without insulin therapy during 36-month follow up, indicating that amniotic membrane stem cell transplantation can improve islet cell function *in vivo* (Liu et al., 2013). Another case report has indicated that co-infusion of HSCs and differentiated insulin-producing cells from adipose tissue-derived MSCs was able to normalize hyperglycemia in a T1DM patient (Dave et al., 2013).

Human ESCs differentiate into endocrine cells, but there is a risk that ESCs promote the development of tumors (Kroon et al., 2008). iPS can be generated from dermal fibroblasts of T1DM patients, and it has been indicated that these cells can be induced into insulin-producing cells, and would enable diagnostic and therapeutic application of basic and translational T1DM research (Maehr et al., 2009; Thatava et al., 2013). However, until now, there are no reports of iPS cells being used to clinically treat T1DM. Human adipose-derived-MSCs can differentiate into insulin-producing cells which were sensitive to glucose *in vitro* (Dave et al., 2012), and human BM-derived-MSCs can differentiate into β cells, which expressed PDX1 and improved hyperglycemia in diabetic mice (Karnieli et al., 2007). One report has shown that human cord blood-derived MSCs are able to differentiate into insulin-producing cells by transduction with non-integrated LV-PDX1 (Boroujeni and Aleyasin, 2013), while HSC transplantation has been shown to be a useful method for treating T1DM patients with autologous HSC transplantation achieving insulin discontinuation (Farge et al., 2010; Snarski et al., 2011).

## Conclusion and future prospects

T1DM therapy using induced β cells from ESCs, iPSCs, and adult stem cells has previously been reviewed (Muir et al., 2014). In the present review, we summarize the pathophysiology of T1DM, and the basic research and clinical studies focusing on developing therapies for T1DM. Cell-based therapy helps prevent the autoimmune destruction of β cells in T1DM, while tissue-derived stem cells such as BM-, adipose tissue-, liver- and pancreas-derived stem cells have the ability to generate insulin-producing cells, and to improve diabetes. BM-derived MSCs inhibit the T cell-mediated immune response against newly-formed β cells, and stem cell therapy may thus be a viable approach to treating T1DM patients. The generation of β cells from various stem cells may help overcome the problem of the lack of donors for islet or pancreas transplantation, and this would be a valuable research topic if these generated β cells were able to avoid immune destruction when the stem cells were allogeneically transplanted. Overall, stem cell research directed at the treatment of T1DM might well also be valuable in regards to other types of DM.

### Conflict of interest statement

The authors declare that the research was conducted in the absence of any commercial or financial relationships that could be construed as a potential conflict of interest.
